# How to develop a theory-driven evaluation design? Lessons learned from an adolescent sexual and reproductive health programme in West Africa

**DOI:** 10.1186/1471-2458-10-741

**Published:** 2010-11-30

**Authors:** Sara B Van Belle, Bruno Marchal, Dominique Dubourg, Guy Kegels

**Affiliations:** 1 Institute of Tropical Medicine, Nationalestraat 155, B-2000 Antwerp, Belgium

## Abstract

**Background:**

This paper presents the development of a study design built on the principles of theory-driven evaluation. The theory-driven evaluation approach was used to evaluate an adolescent sexual and reproductive health intervention in Mali, Burkina Faso and Cameroon to improve continuity of care through the creation of networks of social and health care providers.

**Methods/design:**

Based on our experience and the existing literature, we developed a six-step framework for the design of theory-driven evaluations, which we applied in the ex-post evaluation of the networking component of the intervention. The protocol was drafted with the input of the intervention designer. The programme theory, the central element of theory-driven evaluation, was constructed on the basis of semi-structured interviews with designers, implementers and beneficiaries and an analysis of the intervention's logical framework.

**Discussion:**

The six-step framework proved useful as it allowed for a systematic development of the protocol. We describe the challenges at each step. We found that there is little practical guidance in the existing literature, and also a mix up of terminology of theory-driven evaluation approaches. There is a need for empirical methodological development in order to refine the tools to be used in theory driven evaluation. We conclude that ex-post evaluations of programmes can be based on such an approach if the required information on context and mechanisms is collected during the programme.

## Background

Theory-driven evaluation (TDE) was invented to provide an answer to problems of evaluation approaches that are limited to before-after and input-output designs traditionally used in programme evaluation [1,2]. This was a reaction to the position of Campbell & Stanley [3], who stated that internal validity is the most essential issue in research, and Cronbach's position that evaluation cannot serve policymaking if its external validity is not guaranteed [4]. Chen and Rossi aimed at providing a perspective on evaluation that ensures both types of validity. These authors hold that for any intervention, a programme theory that explains how the planners expect the intervention to work can be described. The programme theory is the often implicit set of assumptions that steers the choice and design of an intervention. Making these assumptions explicit allows to understand what is being implemented and why - it opens up the so-called black box between intervention and outcome. Therefore, the programme theory represents a hypothesis that can be tested and further refined.

**Figure 1:**
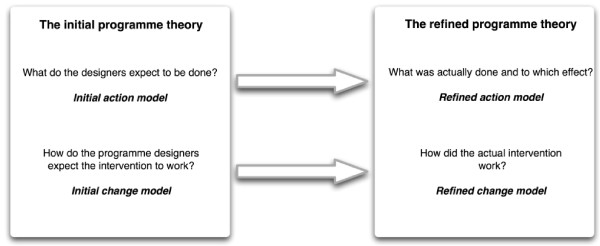
**The components of the initial and the refined programme theory**.

Chen distinguishes the normative from the causal part of the programme theory [1]. The normative theory or *action model *contains the rationale and justification of the programme [5]. It is what programme designers have in mind as assumptions and objectives when designing the programme. In many programmes, these assumptions are not stated explicitly; it is simply assumed that all programme partners share them. Evaluation of the action model describes how exactly the planned intervention has been implemented and allows to check whether an unsuccessful intervention is due to implementation failure or programme design failure. Evaluation of the causal theory or *change model *examines the causal processes and the intervening contextual variables that produce change [5] (see figure 1). In theory-driven evaluation, the results of the evaluation are formulated as an improved programme theory and as such incorporated into the existing body of theoretical and programme knowledge.

Since the 1990s, new developments in the field of theory-driven evaluation include Theory of Change and realist evaluation. The Theory of Change (ToC) approach was developed by the Roundtable on Community Change (Aspen Institute, USA) to evaluate complex community-based change interventions [6]. Mostly applied to programme evaluation, it seeks to establish the links between intervention, context and outcome [7-9] through development and testing of logic models [10].

Realist Evaluation (RE), developed by Pawson & Tilley [11], argues that in order to be useful for decision makers, evaluations need to indicate 'what works in which conditions for whom', rather than merely answering the question 'does it work?'. Realist evaluation aims at identifying the underlying generative mechanisms of the intervention, the "pivot around which RE evolves", [12] - and the influence of context upon the outcomes. It has its philosophical roots in critical realism [13,14]. In this paper, we adhere to the theory-driven evaluation terminology of Chen [15] for reasons of simplicity, acknowledging the wide range of other terms used in Theory of Change and Realist Evaluation.

Theory-driven evaluation somehow disappeared from the radar during the 1990s, emerging again at the European Conference of Evaluation in 2002 [16]. Methodological developments had continued, however, in the field of programme evaluation by authors like Chen [5] and Donaldson [17]. In parallel, ToC and Realist Evaluation were increasingly applied in the evaluation of social care, youth and education policies and programmes [8,18-23].

In health care, there is limited documentation regarding the practical application of research and evaluation designs based on theory-driven evaluation principles. In the domain of health promotion, there are some studies using the ToC approach [21] or Realist Evaluation [24,25]. In the field of health policy and management, papers include [26,27] and [28]. In the domain of medical education, we found two papers ([12,29]). There are even fewer publications focusing on the practical application in public health in low and middle-income countries [21]. These include some research studies in the domain of health service organisation and public health ([30,31]).

This scarcity of theory-driven enquiry in health may be due to various reasons: carrying out a full-blown theory-driven evaluation is resource- and time intensive [2]. Furthermore, guidance on how to apply the principles of theory-driven evaluation in the domain of health systems research is scarce. Indeed, few of the abovementioned papers offer structured approaches to practically carrying out such evaluations or research.

The objective of the evaluation on which we report was not only to assess the intervention, but also to systematically develop a framework for the design of theory-driven evaluations.

We first describe briefly the programme that was evaluated and then present how we developed a 6-step framework for the design of a theory-driven evaluation protocol. For each step, we describe how we applied it during the evaluation. We end by discussing the main challenges we faced, framing our experience in the existing literature.

## Methods/design

We applied the principles of theory-driven evaluation in an ex-post evaluation of one of the programme strategies of the PASSAGE programme, Projet d'Approche Solidaire en Santé Génésique. PASSAGE is a EU funded, three-year intervention aiming at improving the continuity of care in reproductive health in an urban setting in Mopti (Mali), Maroua, (Cameroon) and two districts of Ouagadougou (Burkina Faso), which ran from 2006 to 2009.

The object of the evaluation was the creation of networks between public and private health and social service providers in adolescent sexual and reproductive health. These networks aimed at improving the integration of services and the continuity of care for adolescents.

Based on our experience and existing literature [5,15,17], we developed a six-step framework for the design of theory-driven evaluations in the field of health systems:

- Step 1: Assessing the scope of the evaluation and the appropriateness of TDE

- Step 2: Critical reconstruction of the initial programme theory

- Step 3: Choice of data collection methods & development of tools

- Step 4: Assessing the initial action model: Evaluating relevance of programme design and degree of implementation

- Step 5: Assessing the initial causal model: Establishing the causal mechanisms and contextual factors, and their interactions

- Step 6: Translating findings into the refined programme theory

### Step 1: Assessing the scope of the evaluation: Is TDE needed in order to learn?

Theory-driven evaluation can be quite resource- and time intensive: its scope extends beyond an efficacy/outcome evaluation to include the assessment of the underlying programme theory [32]. Also the need to deconstruct the influence of the context on the intervention and the outcomes requires time [33]. It is therefore important to assess the scope of the evaluation to decide whether a TDE approach is needed. A number of authors have indicated the usefulness of TDE in evaluation of interventions that have attributes of complexity [7,18,34,35]. We argue here that TDE can be used to good effect in case of research or evaluation of an intervention in a complex setting and in case of a new type of intervention, for which the understanding of the causal mechanisms needs to be established.

In practice, the need for a TDE approach for the evaluation of the networking component of PASSAGE was jointly assessed with the commissioner of the evaluation. We found that the evaluation of the networking strategy fulfilled the above indications: it is an intervention in a complex setting where social interaction needs to be mobilized for the intervention to succeed. In order to improve continuity of care for adolescent sexual and reproductive health, PASSAGE intended to create or strengthen linkages between professional and non-professional service providers of different sectors: public and private, medical and social. The creation of networks between these different communities of providers intervening at different levels inside and outside of the health system requires the initiation and maintenance of a social dynamic between them. It could also be argued that the networking component was innovative, and thus requiring in-depth investigation. The creation or promotion of networking is a tested intervention in the field of development (e.g. the creation of national NGO platforms in Sub Sahara Africa) and in public health (e.g. the creation of networks of HIV/AIDS civil society organisations). However, networking has seldom been applied to stimulate (promote) integrated care provision in the domain of reproductive health.

During this step, it was also decided to mainly focus this evaluation on the processes through which the intervention worked (or not). The specific objective was to evaluate to what extent strategies implemented to strengthen networks between actors involved in adolescent sexual and reproductive health (ASRH) contributed to:

- the creation of a common vision on an integrated approach towards ASRH service delivery among the involved service providers

- strengthening the capacities of associations involved in the network and improving their functioning

- an improved integration of services and better continuity of care

- better collaboration between the Regional Directorate of Health, one of the programme's implementing partners, and the networks created or revitalised by the programme.

### Step 2: Critical reconstruction of the initial programme theory

A second step in a TDE evaluation is to make the initial programme theory (PT) explicit, the - often implicit - assumptions of the actors involved in the design and subsequent implementation of the intervention. They include the programme designers and implementation teams in each setting, partners in implementation and the target group, in this case the adolescents. Describing the initial PT explicitly aims at understanding the actors' interpretations of how the intervention is linked to the outcomes through eliciting their assumptions regarding the underlying mechanisms.

Lipsey & Pollard identify different mechanisms to make this PT explicit [36]. First, much relevant information can be gained from the designers and implementers. In this case, the researchers unearth the models that the actors are using to describe and understand the intervention through individual interviews or group discussions. Cole stresses the need to involve the stakeholders and implementers of the interventions during the stage of programme theory development, as one seeks to describe what these actors think compared with what the designers thought [37]. The discrepancy between these views may then be explored as a source of non-implementation [17].

A second source of information for constructing the initial programme theory is relevant theories and current knowledge, such as findings from evaluations of similar interventions. In some cases, the problem situation, the intervention or the policy has been thoroughly researched. The results of these studies can contribute to the formulation of the PT. In other cases, appropriate concepts from disciplines such as cognitive psychology, sociology, etc. can be used [36].

A third approach consists of exploratory on-site research during the various phases of the programme based on observation and inquiry. In all three cases, the feedback of the emerging programme theory to the actors involved is critical, since this allows refining it [36]. In practice, the three approaches are used in combination (see for instance [28]).

When programmes are evaluated, a natural starting point is the logic model presented by the logical framework. In practice, however, the logical framework often offers little information on mechanisms of change. Also, they are usually developed before the start of the programme without much consultation of the implementers or beneficiaries. This lack of useful information often persists, since once the programme starts, there is frequently too little time to build a shared understanding of the logical framework. In such case, the actors typically rely on their own interpretation of the logical framework and this provides the main guidance for implementation [21,34]. If this is the case, one might find that several rival programme theories co-exist and evaluators will need to explore these different interpretations. At the least, they should establish to which degree the initial programme theory was shared by the main actors.

In the evaluation of PASSAGE, we started to draft the programme theory by reviewing the main programme documents, such as the description of the intervention in the programme proposal and the logical framework. We then interviewed the programme coordinator, who also was the main initiator and designer. We explored the literature to frame the programme designers' assumptions against the existing theory.

To structure the initial PT, we used the following elements: the planned intervention and its elements, the planned outputs and outcomes, the context factors assumed to be needed and the processes of change. Table 1 presents the initial PT that was the result of the above process. In a second stage, the programme theory as perceived by each country programme implementation team and by the implementing partners was generated. Divergent interpretations and adaptations to the context, indeed, need to be identified as they may pull the programme's implementation in different directions. To this end, the teams and partners were interviewed. In a third phase, we interviewed adolescents in each site.

**Table 1:** The initial programme theory of PASSAGE

The initial action model (What did programme designers plan to do and expect to attain?)	Bringing together the various actors involved in reproductive health for adolescents in a network increases the access and the utilisation of appropriate social and health services by adolescents and contributes to improving their sexual and reproductive health status.
*The Initial change model *(How was the programme supposed to work based on the programme designers' assumptions?)	The network(ing) contributes to: (1) better knowledge of partners with different backgrounds and their specificities; (2) a growing awareness of a shared vision among partners on adolescent sexual and reproductive health; Knowing each other and each others' specificities and a growing awareness of a shared vision would lead to cooperation and the creation of synergies rather than competition. This in turn would lead to improved ASRH outcomes.

Due to the nature of the intervention, e.g. the large number of actors and associations involved, and the limited time spent at the start of the programme on building a joint understanding of the logical framework, we expected that divergent perspectives on the programme theory would emerge. During the design phase, we decided therefore to describe any such rival PT and compare them in the analysis phase of the evaluation. In practice, we found that the PT of the country programme teams did not significantly differ from the overall PASSAGE PT described in Table 1, but as we will see below, the activities that were actually conducted were different across the sites.

### Step 3: Choice of data collection methods & development of tools

Theory-driven evaluation is essentially method-neutral. Both quantitative and qualitative data collection methods can be used. The choice of data collection methods and the actual data collection process is steered by the aim of the study, its scope and the degree of development of the programme theory: the aim is to collect data to confirm or falsify its different elements and linkages [11].

In this case, we chose for the case study as the overall design, a natural choice for the evaluation of a programme component (in this case 'networking') in which social dynamics are assumed to be important. The case study design allows for exploring a "phenomenon within its real-life context, especially when the boundaries between phenomenon and context are not clearly evident" [38].

Given the main focus of the evaluation on the causative theory, mostly qualitative methods were to be used: both the identification of the key elements of the programme theory as the exploration of underlying mechanisms required interviews and focus group discussions, besides the analysis of progress reports and logbooks.

In practice, this step coincided somehow with step 2, as at that stage, tools were needed for programme document review, the literature review and the interviews with the programme designers. Semi structured topic guides were drafted for these interviews. We discuss the specific data collection issues for step 4 and 5 below.

### Step 4: From initial action model to refined action models: evaluating the relevance of programme design and strength of implementation in the three settings

Once the initial programme theory has been described, the data collection tools designed and the data collected, the programme theory can be used in the next step: the actual assessment of the different dimensions of the programme in function of the actual research questions.

First, the evaluators focus on the action model, describing the programme design on the one hand and its actual implementation on the other. This step assesses the congruency between the planned and the actual intervention and looks at potential issues concerning implementation. It allows distinguishing theory failure from implementation failure [5].

In the case of PASSAGE, we designed the protocol to provide answers to the following questions in each of the study sites:

(1) What was the actual intervention implemented as compared to the planned intervention?

(2) How was the intervention implemented?

(3) What were the results of the intervention?

To this end, the data collection was carried out in the three study settings. In preparation of the fieldwork, a primer in theory-driven evaluation was designed and used for training of the local research teams. Interview guides were drafted, fine-tuned and tested in each field site prior to the actual interviews. A team consisting of 2 evaluators carried out the fieldwork during a 2-week period at each study site.

At the start of the fieldwork, the country-level programme theory was formulated on the basis of interviews with the country programme team members. In a second step, in-depth interviews were carried out with purposively selected key informants in order to obtain information on the actual implementation of the programme, the mechanisms and context (see Table 2). These included representatives of the local authorities and the district and regional health authorities, staff of public and private health facilities, staff and volunteers in youth centres. We also interviewed members of community-based organisations and NGOs involved in adolescent sexual and reproductive health, peer educators and volunteers of school youth groups and neighbourhood youth groups, community and religious leaders. In a third step, focus groups discussions were carried out, the participants of which were divided by sex in separate groups. The age of the participants was between 15 and 24 years. Each group was selected to contain adolescents of different substrata: adolescents from various neighbourhoods, adolescents going to school and being out of school, adolescents following comprehensive education and technical (professional) education, adolescents from private, faith based schools and from public schools.

**Table 2:** Overview of in-depth interviews and focus group discussions

	Mali	Burkina Faso	Cameroun
In-depth interviews	25	24	25

Focus group discussions	1 with 8 male adolescents	1 with 10 male adolescents	1 with 9 male adolescents
	1 with 8 female adolescents	1 with 10 female adolescents	1 with 9 female adolescents

Additional information on programme implementation was obtained by reviewing the progress reports and the logbook kept by the programme coordinator.

### Step 5: From initial change model to refined change models: establishing the causal mechanisms and contextual factors in the three settings

Theory-driven evaluation would not provide an added value compared to result-based (outcome/impact) evaluations if the change model would be left unchecked. This step traces the mechanisms that link the actual intervention to the actual outcomes. By mechanism, we understand the causal pathway that is made up by the interplay between intervention, actors and contextual conditions. This interplay may consist of both linear relations and feedback loops that ultimately lead to change.

The evaluation of the change model answers three questions: (1) What kind of relationships exist between actual intervention and outcome?; (2) Which intervening factors could be mediating the effect of the intervention on the outcome variables? and (3) Under what contextual conditions will the causal relationship be facilitated or inhibited? [5].

**Figure 2:**
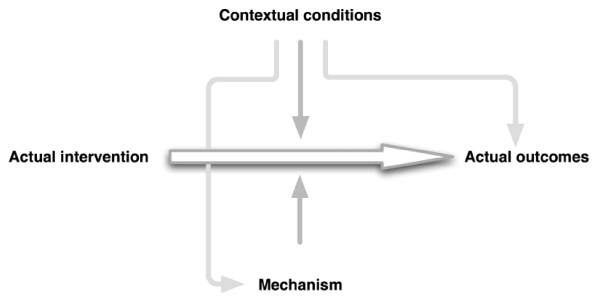
**The change model**.

### The actual intervention

In the case of the PASSAGE evaluation, we proceeded by first describing the networking component of the programme as it was actually implemented on the ground in each site. We found that the actual networking component differed across the sites (Table 3). Also the speed of their development varied. In Mopti (Mali), it took some time to warm NGOs to the idea of a reproductive health network and during the evaluation, network members were still in the process of exploring the possibilities.

**Table 3:** The networking activities in the 3 sites

Mali	The project team decided to strengthen the functioning of an existing NGO network that grouped HIV/AIDS NGOs of the region. This network was in a fragile state due to lack of leadership. The team decided to expand its membership to NGOs working in sexual and reproductive health.
Burkina Faso	Networking efforts were focused on the improvement of access to ASRH services through filling in gaps in the referral chain. Two networks were launched: REPERE (*Réseau des Personnes Référentes*) and RESCOPE (*Réseau des Structures Communautaires pour la promotion de la Paire-Education*). REPERE brings together individuals, working in both public health structures and private non-for-profit associations, who volunteer to act as an entry point for information for adolescents in need of ASRH services. Volunteers can be contacted by adolescents when in need. RESCOPE and REPERE work in tandem: peer educators of different youth associations provide information themselves or could refer adolescents in need of youth friendly service providers.

Cameroon	Three networks were launched: one bringing together peer educators of existing school clubs that were working on ASRH and HIV/AIDS prevention, one resource persons network, and a network of NGOs/CBOs working on HIV/AIDS prevention.

### The results

In a second step, we assessed the results of the intervention.

- In general, we found that the intervention in the three settings resulted in closer collaboration between partners who before the programme were only loosely connected.

- We found that the exchange between different NGOs during network meetings resulted in several activities that brought together NGOs and technical services or formal health structures. In Burkina Faso, for instance, the networks launched by the project have resulted in a better ad hoc referral between ASRH curative and preventive services. Meetings were organized that bring together all actors of both public and private non-for-profit sector. At the Cameroon intervention site, all youth volunteers active in reproductive health and working in schools were brought together, creating linkage and exchange between adolescents of different denominations and backgrounds.

- Our data indicates that the networking strategy led to increased organisational learning through exchange of information, expertise and material resources.

- It also led to an analysis of the offer of care and some remedial action. The health professionals became aware of lacunae in the provision of and access to ASRH services and that actions were taken that improved the continuity of care for adolescents.

- We also found that the DRS, who according to the plan was to take up the coordinator role, did support the networking activities in all three settings but did not fully take up the role of coordinator.

### Mechanisms

In a third step, we sought clues and information for these mechanisms during the in-depth interviews and observations. To do so, we included questions covering the following themes: the process of networking (the process of setting up networks or revitalisation of existing ones, network members and connections, activities organised by the networks, etc.); results of activities conducted by the network (sharing of knowledge, dialogue, improved coordination under the aegis of the regional health authorities, etc.), appropriateness of the networking strategy to the site context, and the sustainability of the networks.

We found that important factors were: (a) perceived individual and organizational opportunities and (b) an individual or organizational awareness of the lacunae in ASRH service delivery leading to a commitment to improve ASRH services. Individuals and organizations want to participate actively in a network when they perceive that this is of added value to their functioning. Network actors joined a network because it enhances their organizational visibility, to liaise and learn from other resource persons and organizations in the field (as most organizations are not specialized in ASRH and recognize that they are in need of additional expertise), to have access to information and training and, last but not least, to have access to additional funding opportunities.

### Context

Fourth, we set out to describe the influence of the context. The literature shows that contextual conditions that facilitate or inhibit processes of change entail institutional arrangements, stakeholders' and target groups' attitudes and behaviours, and geographical and socio-cultural factors, either at meso- or macro level. During the analysis, two conditions emerged from the data. These were related to the networking process and to the relationship between networking as an intervention and the outcome. An example of the latter is the urban setting of the project, which facilitates communication between network members. We found that the following contextual conditions are related to the networking process itself:

- The competition context determines the degree of the net benefits to networking for the actors concerned. In a highly competitive environment, where NGOs have to compete for scarce resources, it might well be that networking, and particularly the sharing of information with other NGOs in the same field, might be perceived as detrimental to the organization.

- The commitment of the Regional Directorate of Health to be part of the network, to coordinate (stimulate) it and to oversee the private-not-for-profit sector not only depends on the benefits of this role for itself. It requires resources to do so, and we found that in all three settings, the DRS currently lacks the necessary financial resources, both financial, human resources and time, to take up this role. Furthermore, given the resource poor context, taking on a stewardship role might prove not to be beneficial as this could have negative financial implications. The private-non-for-profit sector could ask for financial support for activities that are of mutual benefit.

- For the DRS to take up the coordination role in a non-hierarchical structure such as the PASSAGE networks, it has to be accepted by the non-for-profit sector as the steward in adolescent sexual and reproductive health. In the Burkina Faso setting, private non-for-profit actors saw the benefit of working alongside the DRS for medical supervision and technical assistance. This was not the case in the other settings.

We summarised the resulting analysis of this step in a diagram of the causal pathways, which was validated through discussion with the programme partners during the fieldwork and analysis phase.

**Figure 3:**
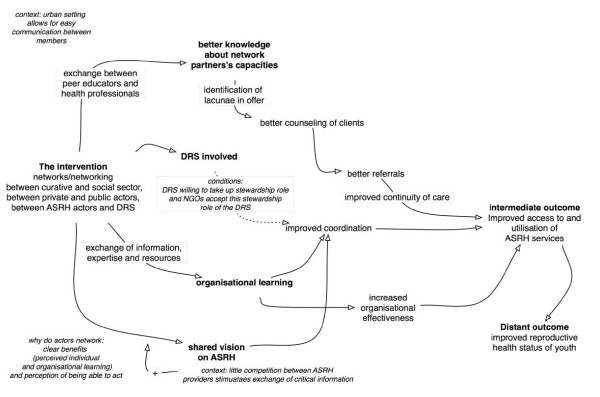
**The causal web**.

### Step 6: Generalization to the level of a refined programme theory

Theoretically, TDE yields results that have a higher external validity, because it ends with a refined programme theory that explains under which conditions and how the results were obtained. However, the literature does not provide us with much practical lead on how to generalize from particular evaluation findings. This is partly because of the non-linear, creative nature of theory constructions, where one goes back and forth between intuition and data, and between induction and deduction [39], a process that is hard to formalise.

In the case of evaluations, the refined PT should ideally make sense to the users of the evaluations and meet the purpose of the evaluation as defined by its commissioners. Furthermore, it needs to be able to serve as the starting point of evaluations of similar interventions, thus adding to an ever-increasing knowledge base regarding a particular intervention [39,40]. To this end, it should be formulated so as to explain not only whether the intervention works, but also how, for whom and in which context. In the case of PASSAGE, we ended by formulating the refined programme theory in a narrative form (Table 4).

**Table 4:** The refined PT

The refined programme theory of Passage	Bringing together the various actors involved in reproductive health for adolescents in a network can increase the access and the utilisation of appropriate social and health services by the adolescents and contributes to improving the reproductive health status of the adolescents if (1) it succeeds to bring together actors that cover the whole range of services required by adolescents, (2) creates a shared vision and (3) leads to integration of all ASRH services.
	Active networking contributes to: (1) a shared awareness that the current services are ineffectual because of gaps and redundancies in the provision (2) better knowledge of partners with different backgrounds and thus to better informing adolescents and to more effective referrals, which in turn contributes to better continuity of care (3) a shared vision among partners on ASRH, which contributes to better coordination and integration of services (3) organisational learning, which enhances coordination and quality of care and services.
	The underlying processes include increasing *linking *social capital and organisational social capital. The latter strengthens the relations between organisations, the former stresses the relations between organisations and public authorities. Partners need to perceive a win-win situation to continue to be active members and to experience a feeling of ownership. Existing networks can be mobilised to take on new tasks, inactive networks can be revitalised (but this requires more time and inputs), or completely new networks can be set up (the longest route).

## Discussion

In this paper, we identified conditions that can be used to decide whether a theory-driven evaluation would be indicated. We discussed how the protocol was constructed around 6 steps that systematically apply the principles of theory-driven evaluation to an ex- post evaluation, presented the challenges and gave examples of the findings that emerged from the actual evaluation at each step.

During the design and implementation phase, we were confronted with several challenges. First, we faced the challenge of the variable and, at times, too vague terminology used by theory-driven evaluation experts and methodologists. Each major school develops its own terminology (see for instance 'middle range theory' [11], theory of change [6] or programme theory [15]; or normative and causative theory [15] versus action and causal model [5]. In many papers, the different approaches of theory of change, theory-driven evaluation and realist evaluation are somehow mixed up and terms of different schools are used interchangeably (see for instance [18,19,41].

The issue of identifying 'rival' programme theories provides a good example of the limited published guidance. Rival PT are the result of actors' different viewpoints and positions vis-à-vis the intervention (for instance: initiator and designer versus implementer versus adolescents; the perspective from the South versus from the North). It is therefore important to identify whether any rival PT were held and how they influenced the programme. During the design phase, we realised that the heads of the country teams could have other interpretations of the goals and strategy of PASSAGE on the basis of their different professional backgrounds and experiences or personal preferences. We found some guidance in the literature: if different actors are gathered to discuss the programme theory at the programme start-up phase, the role of the evaluator will be one of negotiator between groups in an - in essence - political process [21,42]. If the evaluator is involved in the building of the M&E system at the beginning of the programme, clear responsibilities between the programme coordinator and the evaluator need to be delineated to avoid a blurring of roles between them [21]. As mentioned above, in PASSAGE, we decided to maintain any such rival theory as an alternative hypothesis to be tested during the analysis.

Other challenges relate to the application of the TDE approach to ex-post programme evaluations. In essence, routine M&E systems of programmes do not monitor the contextual conditions that may be important, nor do they provide information that could allow identifying the underlying mechanisms. Combined with the issue of recall bias, this presents major challenges. One could argue that TDE could still be applied if during the evaluation, the change processes are explored in a joint reflection process where all actors join in, for instance during an end-of-programme closure workshop. We would tend to believe that such discussions would yield interesting information but not allow for a robust evaluation. It could thus be argued that ex-post evaluations of Log Frame based programmes are not possible, or at least that a complete application in its full scope is not feasible. Only if appropriate monitoring systems are built in the programme can information to identify mechanisms and contextual conditions be available at the end of a project.

Finally, we faced some more general challenges. First, there is the issue of the role and the skills of the evaluators. Development intervention evaluators are commonly driven towards establishing the outcomes of the programme and focus on changes within the target group of the intervention. Theory-driven evaluation requires additional training or thorough briefings to modify the evaluator's point of view from an exclusively results-driven focus (i.e. as needed in effectiveness evaluations) to a process-oriented focus that is needed for theory-driven evaluation. We found that theory-driven evaluation teams ideally have broad competencies, experience and expertise that allow for the identification of mechanisms of change and of the relevant contextual factors.

Second, it is often argued that TDE is time consuming [2]. In practice, we found that a TDE approach should not necessarily take more time than regular evaluations of similar multi-country programmes. In the case of PASSAGE, the preparation of the evaluation by the political scientist took about 2 weeks time, including the design of the protocol and the primer on TDE used in the training of the anthropologists. The fieldwork took the evaluation team consisting of one political scientist and one anthropologist 2 weeks per site. The analysis was based on site reports written by the anthropologists (2 weeks per site) and the comparative analysis took 4 weeks, including the draft of the final report.

A third general challenge is the issue of complexity. One major setback and perhaps also a reason why there is currently not an abundance of theory-driven evaluations of public health interventions, is the challenge that 'complexity' presents to the causal attribution. Whereas we argued above that theory-driven evaluation designs are appropriate for complexity, the very complex nature of the programmes stands in the way of an easy assessment of effectiveness and of underlying mechanisms: the outcomes of complex interventions can be attributed to a number of determinants, only some of which are influenced by the intervention. It would however be a mistake to adopt the standards of strength of evidence from the biomedical world in assessing the value of findings of evaluations of complexity. As Pawson & Tilley argue convincingly, in such cases, theory-driven evaluation will aim at offering plausible explanations, not probabilistic statements [11].

## Conclusions

To conclude, the theory-driven evaluation approach holds much promise for relevant learning from public health interventions and programmes, but there still is a need for methodological development for practical use. Ex-post evaluations of programmes can be based on such an approach if the required information on context and mechanisms is collected during the programme.

TDE inevitably requires an element of practitioner "craft", involving judgment and creativity based on broad theoretical induction and background, and experience. Ways to reduce the danger of arbitrariness, unwarranted subjectivity and superficiality include (1) introducing the theory-driven perspective from the start of the programme, and (2) documented critical exchanges among TDE practitioners on how they deal effectively with vagueness and conceptual ambiguity.

## Competing interests

The authors declare that they have no competing interests.

## Authors' contributions

SVB contributed to the design of the evaluation protocol, the actual evaluation, the analysis of findings and the writing of the manuscript. BM contributed to the protocol development and the writing of the manuscript, DD participated in the protocol development and manuscript revision, and GK in manuscript writing and revision. All authors read and approved the final manuscript.

## Pre-publication history

The pre-publication history for this paper can be accessed here:

http://www.biomedcentral.com/1471-2458/10/741/prepub
